# FBG and BOTDA Based Monitoring of Mine Pressure Under Remaining Coal Pillars Using Physical Modeling

**DOI:** 10.3390/s24217037

**Published:** 2024-10-31

**Authors:** Dingding Zhang, Zhi Li, Yanyan Duan, Long Yang, Hongrui Liu

**Affiliations:** 1 College of Energy Engineering, Xi’an University of Science and Technology, Xi’an 710054, China; lizhi@stu.xust.edu.cn (Z.L.); 23203226082@stu.xust.edu.cn (L.Y.); heyyyfy@163.com (H.L.); 2 Key Laboratory of Western Mine Exploitation and Hazard Prevention, Ministry of Education, Xi’an University of Science and Technology, Xi’an 710054, China; 3 China United Northwest Institute for Engineering Design and Research Co., Ltd., Xi’an 710076, China; dyy-kpqn@163.com; 4 China Energy Shenhua Shendong Coal Group Co., Ltd., Yulin 719315, China

**Keywords:** FBG, BOTDA, remaining coal pillar, mine pressure, physical model experiment

## Abstract

Strong mine pressure often emerges when the working face of the lower coal seam in a closely spaced coal seam system passes through the remaining coal pillar in the overlying goaf. This study investigates the law of overburden movement and the manifestation of mine pressure during mining under the remaining coal pillar. A physical model measuring 2.5 × 0.2 × 1.503 m is constructed. Fiber Bragg grating sensing technology (FBG) and Brillouin optical time domain analysis technology (BOTDA) are employed in the physical model experiment to monitor the internal strain of the overlying rock as the working face advances. This study determines the laws of overlying rock fracture and working face pressure while mining coal seams beneath the remaining coal pillar. It analyzes the relationship between the pressure at the working face and the strain characteristics of the horizontally distributed optical fiber. A fiber grating characterization method is established for the stress evolution law of overlying rock while passing the remaining coal pillar. The experimental results indicated that the fracture angle of overlying rock gradually decreases during the mining stage through and after the coal pillar. In the mining stage through the coal pillar, the cycle pressure step distance of the working face is reduced by 33.3% compared to the stage after mining through the coal pillar. Initially, the strain pattern of the horizontal optical fiber is unimodal when pressure is first applied to the working face, and it transitions from unimodal to bimodal during periodic pressure. The peak value of fiber Bragg grating compressive strain and the range of influence of advanced support pressure are 3.6 times and 4.8 times, respectively, before passing through the remaining coal pillar. Finally, the accuracy of the FBG characterization method is verified by comparing it to the monitoring curve of the coal seam floor pressure sensor. The research results contribute to applying fiber optic sensing technology in mining physical model experiments.

## 1. Introduction

China is a country whose energy structure is dominated by coal. Coal production has reached 4.71 billion tons, accounting for more than 50% of total energy consumption. With the continuous mining of coal resources, the single coal seam at the top of most mines has been essentially exhausted, and the lower coal seams have been mined successively [1]. In the coal mining process, due to the influence of mining technology, geological conditions, equipment levels, and other factors, a significant number of remaining coal pillars are left in the goaf after the top coal is mined. This results in severe mine pressure when mining under the remaining coal pillars in the lower coal seams. Currently, considerable progress has been made in researching the behavior of mine pressure and control technology in the mining of single coal seams, and the dynamics of roof activity and overburden stress distribution in the mining process of different thickness coal seams have been clarified. The mine pressure behavior in close-distance coal seam mining is more evident than in single coal seam mining. When the working face of the lower coal seam passes through the remaining coal pillars in the overlying goaf, it often encounters intense pressure, overall roof cutting, support dynamic load, and other dynamic pressure phenomena, which seriously endanger the safety of personnel and mining equipment. It is imperative to study the behavior of mine pressure when close-distance coal seam mining intersects the remaining coal pillars.

The physical model experiment based on similarity theory involves reducing the engineering problem to a laboratory model relying on a specific similarity ratio, obtaining the relevant parameters of the model experiment through various monitoring methods, and extrapolating the rules to the prototype [2]. Physical model experiments can intuitively and qualitatively simulate the pressure behavior of the working face during coal mining and establish the internal relationship with the law of overburden rock migration. This approach can effectively address the deficiency of theoretical research on overburden rock migration, thus solving the black box problem and becoming an essential test method to study the law and mechanism of mine disasters. In physical model experiments, the deformation monitoring of the physical model primarily uses total stations, dial gauges, strain gauges, and others, while stress monitoring mainly uses floor pressure sensors, pressure boxes, and pressure measuring tubes, among other sensors. At the laboratory scale, these methods suffer from low testing accuracy, significant human error, and the inability to test the internal deformation of the model. Therefore, applying optical fiber sensing technology, which offers high precision, good reliability, and internal strain measurement of the monitored body, is an inevitable process in scientific research and development.

Optical fiber sensing technology was developed alongside the advancements in optical fiber communication technology in the 1970s. It represents a new type of sensing technology that uses light waves as the carrier and optical fibers as the medium to sense and transmit the measured signals [3]. In the 1990s, this technology was applied to investigate both micro and macro processes of material failure to further understand its physical and mechanical properties and the evolution of fractures and cracks [4,5]. In 2004, Chai [6] first embedded an optical fiber sensor into the rock strata of a physical model to simulate the rock strata movement caused by mining and proposed the theory and technology of using optical fiber sensing to monitor rock mass deformation. Then, fiber grating sensing technology and Brillouin optical time domain analysis (BOTDA) were employed to characterize the three-zone partition of overburden rock, analyze pressure laws, and study rock movement laws in physical model experiments [7,8]. Juraszek [9] embedded specially designed optical fiber Bragg grating (FBG) sensors into physical models to monitor the strain values in reinforced road structures in areas affected by mining activities. Piao [10] explored the relationship between overburden deformation and implanted FBG sensors during the physical model experiment of backfill mining, successfully recognizing rock stability. Zhang [11] quantitatively assessed the deformation behavior of similarity mining models using PPP-BOTDA, verifying that distributed fiber optic sensing technology is an effective method for monitoring formation strain in coal mining physical model experiments, especially when model deformation is minimal. Liu [12] analyzed the coupling relationship between distributed optical fiber and rock mass and explored the internal relationship between the spatial and temporal evolution characteristics of vertical zoning of mining overburden and the peak point position of optical fiber strain data. Zhang [13] established a distributed fiber optic characterization model for the mountain rotation process caused by coal seam mining and divided the stages of mountain rotation deformation. Hu [14] utilized distributed fiber sensing technology to study the development height of the water-conducting fracture zone in the physical model experiment, finding that the monitoring results show that the evolution stage of the water-conducting fracture zone is a goaf-caving zone-fracture zone. The existing research mainly focuses on the coupling relationship between the optical fiber sensor and the physical model, the corresponding relationship between optical fiber test data and the rock-breaking form, and the testing of the internal stress of the model. Both FBG and BOTDA enable measurement inside the model; FBG offers the advantage of high-precision local strain measurement of the model, while BOTDA provides distributed measurement and positioning of strain [15,16].

In this research, the benefits of FBG and BOTDA are integrated and applied to a physical model experimental study of mining under a remaining coal pillar in the lower coal seam working face of the close-range coal seam. The fracture characteristics and pressure laws of overlying rock are analyzed when the working face passes through the remaining coal pillar. The joint characterization method of FBG-BOTDA for the migration characteristics of overlying rock is explored, and the pressure mechanism of the working face under the influence of the remaining coal pillar is revealed, providing an experimental basis for the safe mining of the lower coal seam working face under the remaining coal pillar.

## 2. Experimental Design of Physical Model Based on Fiber Optic Sensing

### 2.1. Fiber Optic Sensing Principle

1. FBG Sensing Principle

Fiber Bragg grating (FBG) employs internal writing or lasers to create periodic defects in optical fibers, changing the refractive index of the core region. When external parameters such as temperature and stress change, the refractive index of the grating is altered, causing the sensor wavelength to drift. Strain and temperature can be determined by detecting this wavelength drift [17]. Changes in external strain and temperature both influence the wavelength of reflected light, which can be described as follows:

(1) $$∆\varepsilon =\left({∆\lambda }_{B}-K_{T}∆T\right){/K}_{\varepsilon }$$

where Δ*λ_B_* is the change in the center wavelength of the fiber grating, nm; *K_T_* is the temperature sensitivity coefficient; Δ*ε* and Δ*T* are the strain and temperature changes of the grating; *K_ε_* is the strain sensitivity coefficient. For unpackaged bare fiber Bragg gratings, *K_T_* is usually set to 0.794 pm/°C and *K_ε_* to 0.758 pm/με at room temperature. However, fiber Bragg grating sensors with different packaging structures exhibit varying *K_T_* and *K_ε_* values, usually calibrated using water bath heating and tensile experiments. When using Formula (1), their calibrated values should be selected. This method facilitates the calculation of external strain Δ*ε* and is based on the theory of fiber coupling modes, measuring the wavelength change of reflected light.

2. Principle of BOTDA

**Figure 1:**
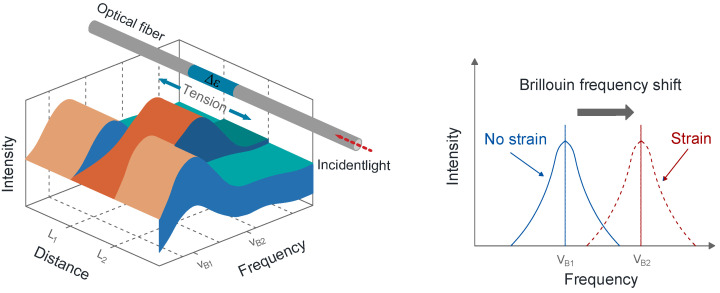
Monitoring principle of BOTDA.

The Brillouin scattered light generated during light propagation in optical fibers results from nonlinear interactions between incident light and phonons thermally excited within the light propagation medium. Changes in the external environment of a specific part of the optical fiber lead to variations in the strain or temperature of the fiber, thus altering the Brillouin frequency of that segment [18,19,20]. Figure 1 illustrates the monitoring principle of BOTDA.

A relationship exists between the Brillouin frequency shift (BFS) *ν_B_* and temperature *T* or strain *ε*, as expressed in the following equation [21]:

(2) $$V_{B2}=V_{B1}+C_{T}(T-T_{0})+C_{\varepsilon }\Delta \varepsilon$$

where V*_B_*_2_ is Brillouin scattered frequency shift with strain at a specific temperature *T*; *V_B_*_1_ is Brillouin scattered frequency shift without strain at temperature *T*_0_; *C_T_* is the temperature sensitivity coefficient of the fiber; *C_ε_* is the strain sensitivity coefficient of the fiber; *T*_0_ is the initial temperature.

During the physical model experiment, a constant ambient temperature must be maintained to prevent changes in ambient temperature from affecting the fiber wavelength and Brillouin frequency.

### 2.2. Physical Model Experiment Design

The Huojitu well of the Daliuta Coal Mine is located south of Daliuta Town, Shenmu County, Shaanxi Province. It primarily extracts the 1^−2^ and 2^−2^ coal seams. The coal seams are buried at depths ranging from 80 to 150 m, with an average interval of 30 m. The characteristics of the coal seams include a shallow burial depth, a small base load ratio, loose overlying rock, and a significant number of section coal pillars with widths of 15 to 30 m left in the goaf of the upper coal seam, which are staggered with the working face of the lower coal seam. There are differences between shallow-buried coal seams and conventionally buried coal seams regarding overlying rock movement, roof-breaking characteristics, and mine pressure manifestations after mining. During the period when the 22206 working face of the 2^−2^ coal seam passed through the coal pillar left by the upper coal seam, there was a strong dynamic load mine pressure phenomenon characterized by extensive roof cutting, and most of the support was damaged, which severely impacted the mine’s safe and efficient production.

**Figure 2:**
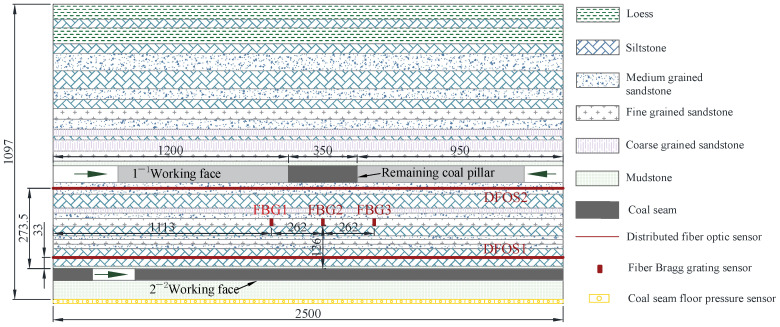
Physical model lithology distribution and sensor layout.

The subject of this physical model experiment is the 22206 working face, where the average burial depth of the 2^−2^ coal seam is 97.9 m and the thickness is 4.5 m. The distance between the 1^−2^ and 2^−2^ coal seams is 29.6 m. The experiment was conducted on a flat model frame with a width of 200 mm, and the physical model construction length was 2500 mm. Due to the fixed size of the experimental model frame, the height of the physical model *lm* was ultimately set to 1097 mm to simulate the prototype mining process more realistically. Based on the principle of similarity, the similarity coefficient of the model can be determined (m is the model, p is the prototype) [22]. Therefore, the geometric similarity coefficient: *C_l_* = *l_p_*/*l_m_* = 100, the displacement similarity coefficient: C_s_ = *μ_p_*/*μ_m_* = *C_l_* = 100, *lp* is the prototype depth, and *μ_p_* and *μ_m_* are the displacement values and model displacement value, respectively. The density similarity coefficient *ρ_γ_* can be obtained using the average density *ρ_p_* of the formation, which is 2600 kg/m^3^, and the density *ρ_m_* of similar materials, which is 1600 kg/m^3^, resulting in *C_ρ_* = *ρ_p_*/*ρ_m_* = 1.56. The stress similarity coefficient is *C_σ_* = *C_l_* × *C_ρ_* = 156. The materials utilized to construct the physical model consist of a mixture of river sand, gypsum, mica powder, fly ash, and water. The mass ratios of similar materials corresponding to different lithologies are detailed in Table 1, and the lithological distribution and sensor layout of the physical model are illustrated in Figure 2.

The monitoring system for physical model experiments consists of a BOTDA monitoring system, an FBG monitoring system, and a pressure acquisition testing system, which respectively track the distributed strain, local strain, and coal seam floor pressure values in the model. The layout of these systems is illustrated in Figure 2. The BOTDA monitoring system consists of the NBX-6055 fiber optic stress analyzer manufactured by Neubrex and distributed fiber optic sensors (DFOS). The monitoring range of the NBX-6055 fiber optic stress analyzer extends from 0 to 25 km, with a minimum spatial resolution of 50 mm. For the test, settings for the NBX-6055 optical fiber stress analyzer are as follows: spatial resolution of 50 mm, sampling interval of 10 mm, sweep frequency range of 10,650–11,250 MHz, average count of 2^16^, and a measurement frequency scan step of 2 MHz. The distributed fiber optic strain sensor utilizes a 2 mm diameter, tightly fit single-mode optical cable with a strain sensitivity coefficient of 0.049 MHz/με. Two horizontally distributed fiber optic strain sensors, labeled DFOS1 and DFOS2, are positioned in the physical model at distances of 33 mm and 273.5 mm from the 2^−2^ coal seam, respectively. During the physical model paving process, distributed fiber optic sensors are embedded in the model, and a certain amount of prestress is applied at both ends of the fiber optic cable to keep it in a stretched state. The FBG monitoring system comprises the MOI-SI225 fiber Bragg grating demodulator manufactured by Micron Optics and fiber Bragg grating strain sensors. The MOI-SI225 fiber grating demodulator achieves a wavelength recognition accuracy of 1 pm and a maximum data acquisition frequency of 5000 Hz, and offers four optical channels. During the test, the data acquisition frequency is set at 1 Hz. The fiber optic grating strain sensor, encased in polypropylene, measures 40 mm long with a calibrated strain sensitivity coefficient of 1 με/pm. Three fiber optic grating strain sensors, numbered FBG1, FBG2, and FBG3, are vertically buried at a distance of 126 mm from the coal roof in the middle of the model, with a horizontal spacing of 262 mm. FBG1 is positioned 87 mm from the left boundary of the remaining coal pillar, FBG2 is at the centerline of the remaining coal pillar, and FBG3 is 87 mm from the right boundary. The pressure acquisition test system consists of an instrument and coal seam floor pressure sensors (CFP). Within the 2^−2^ coal seam floor, 50 coal seam floor pressure sensors, each 50 mm in width, are arranged to monitor the floor pressure while mining the 2^−2^ coal seam. The sensors are numbered from CFP1 to CFP50.

### 2.3. Experimental Process

**Figure 3:**
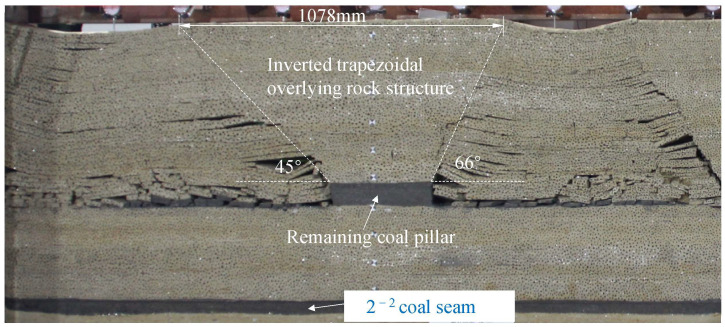
The collapse form of overlying rock after 1^−2^ coal seam mining.

Initially, coal seams 1^−2^ are extracted from both sides of the physical model towards the center, leaving no boundary coal pillar at the end of the model. The cumulative mining distance in the left goaf is 1200 mm, with a mining step spacing of 50 mm, totaling 24 excavations. The cumulative mining distance in the right goaf is 950 mm, and the mining step spacing is 50 mm, resulting in 19 excavations. After mining, a 350 mm section of coal pillar remains in the goaf. An inverted trapezoidal overburden structure forms above the remaining coal pillar. The left collapse angle is 48°, and the right collapse angle is 66°. The longest side of the inverted trapezoidal structure, unaffected by mining, measures 1078 mm. The overburden above the remaining coal pillar tends to tilt to the left, as depicted in Figure 3.

After mining the upper coal seam, the lower coal seam 2^−2^ is extracted, leaving a 200 mm boundary coal pillar. The mining direction moves from left to right, passing through the left coal pillar. The mining step distance is 50 mm, with a total of 45 excavations and a cumulative mining distance of 2300 mm.

## 3. The Deformation Law of Overlying Rock During Mining Through the Remaining Coal Pillar

**Figure 4:**
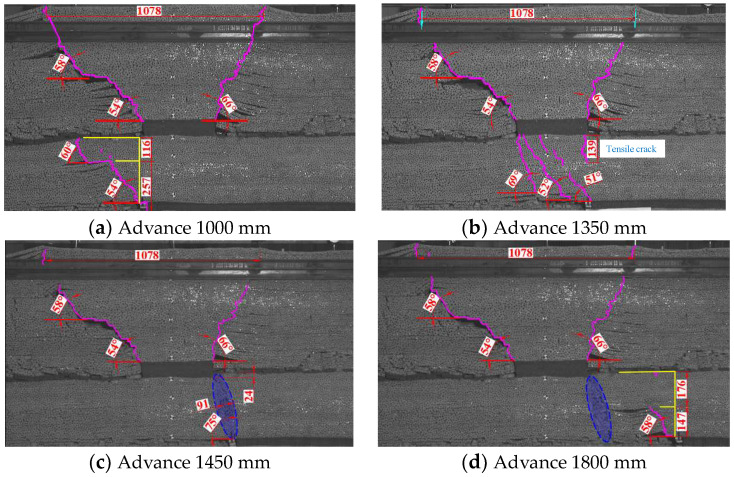
Characteristics of overlying rock collapse as the working face advances.

The uneven load distribution on the roof of the lower coal seam, resulting from the remaining coal pillar, will cause different deformation patterns of the working face and overlying rock during the mining process compared to scenarios without a remaining coal pillar. The mining through the remaining coal pillar is divided into three stages: before, during, and after mining through the coal pillar. In the stage before mining through the coal pillar, the working face of the lower coal seam advances to 500 mm, and the main roof broke for the first time. The roof collapse height is 157 mm, with a collapse angle of 49° on the mining side and 52° on the cutting side. As the working face progresses, the main roof undergoes periodic fractures, and the water-conducting fracture zone develops upwards, forming a continuous fracture with the 1^−2^ coal goaf, causing the overlying rock strata to collapse towards the goaf. The working face advances to 1000 mm, located below the boundary of the remaining coal pillar, where the primary roof broke in the third cycle. The roof breaks and rotates behind the working face, and the collapsed overlying rock exhibits a double collapse angle. The collapse angle of the overlying rock above the coal seam floor from 0 to 257 mm is 54°, and the collapse angle between 257 mm and the 1^−2^ coal seam floor is 60°, as shown in Figure 4a.

When the working face advances beyond 1000 mm, it enters the mining stage through the coal pillar. When the mining passes through a remaining coal pillar of 100 mm, the main roof undergoes its fourth cycle collapse. The overlying rock fractures above the working face extend to the boundary of the remaining coal pillar and connect with earlier fractures to form a through fracture. The collapse angle on the mining side is 69°, with no significant movement in the remaining coal pillar and the intact inverted trapezoidal overlying rock structure above it. As the working face progresses, the cracks above continue developing towards the floor of the remaining coal pillar. Due to the intact and damaged overlying rock above the remaining coal pillar; these cracks no longer connect as through cracks to the goaf of the upper coal seam. Therefore, during the mining process through the remaining coal pillar, the collapse angle of the overlying rock above the working face decreases from 69° to 51°, as depicted in Figure 4b. At this point, the remaining coal pillar and its intact inverted trapezoidal overlying rock structure begin to turn and sink towards the goaf, with the left side of the structure descending by about 5.6 mm and the right side rising by 4 mm. A tensile crack forms on the floor at the right boundary of the remaining coal pillar, extending approximately 139 mm.

After the working face advances beyond 1350 mm, it enters the stage after mining through the coal pillar. When the mining activity of the working face exceeds the remaining coal pillar by 100 mm, the main roof collapses in the seventh cycle, and the fissures in the overlying rock of the lower coal seam and the goaf of the upper coal seam form through fissures, causing the entire inverted trapezoidal overlying rock structure above the remaining coal pillar to sink, with maximum subsidence of 25 mm. The collapse angle of the overlying rock increases to 75°, as shown in Figure 4c. As the working face continues to advance, moving away from the remaining coal pillar, it is less affected by the concentrated load of the coal pillar. The collapse angle of the overlying rock gradually decreases, as shown in Figure 4d.

The pressure step distance and overburden collapse angle during the advancement of the working face are detailed in Table 2. The working face underwent 12 cycles of pressure, with an initial pressure step distance of 500 mm, a maximum of 200 mm, and a minimum of 100 mm. The average cycle pressure step distance before reaching the influence area of the remaining coal pillar is 168 mm. After surpassing the average cycle of the remaining coal pillar, the pressure step distance decreases to 112 mm. During the mining stage through the coal pillar, the pressure step distance at the working face is reduced by 33.3% compared to before passing through the coal pillar. As the working face traverses the equilibrium point of the remaining coal pillar and the complete inverted trapezoidal overlying rock structure above it, the integrity of the upper structure deviates towards the side with the smaller collapse angle, and a downward tensile crack develops at the boundary line between the front of the working face and the remaining coal pillar. When the working face exceeds the remaining coal pillar, a “tension shear” crack zone inevitably forms between the mining side of the remaining coal pillar and the working face, causing the roof to become highly fragmented.

## 4. Fiber Optic Characterization of Overburden Fracture Characteristics

**Figure 5:**
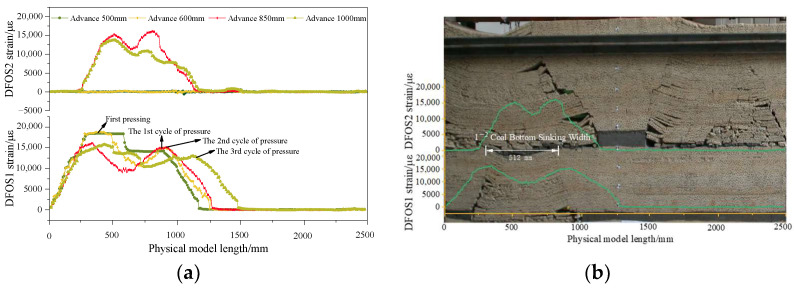
DFOS strain curve before mining through coal pillar. (**a**) Advance from 500 to 1000 mm; (**b**) Overlying rock collapse characteristics when advancing 850 mm.

The DFOS strain curve and the collapse characteristics of the overlying rock before passing through the coal pillar are illustrated in Figure 5. The working face advanced 500 mm, causing the main roof to break for the first time. DFOS1, within the model width range of 0–1150 mm, was in a tensile state, exhibiting a single peak strain curve with a peak tensile strain of 10,609 με. The top plate of coal seam 2^−2^ did not collapse to the floor of coal seam 1^−2^, so the strain on DFOS2 remained unchanged. When the working face advanced to 650 mm, the main roof broke during the first cycle, transitioning the DFOS1 strain curve from a single to a double peak. The strain peak on the cutting eye side was higher than on the working face side, with tensile strain peaks of 18,759 με and 14,997 με, respectively. The subsidence of the 1^−2^ coal floor caused DFOS2’s strain curve to exhibit a distinct bimodal structure, as depicted in Figure 5b, with peak strains of 15,310 με on the cutting side and 16,079 με on the working face side. When the working face advanced to 850 mm, the second cycle of the main roof broke, and the rock block broke and rotated, causing the mining cracks in the top plate of coal seam 2^−2^ to connect with the goaf of coal seam 1^−2^, leading to extensive subsidence of the overlying rock. The tensile range of DFOS1 moved forward in sync with the mining direction of the working face, with the peak strain on the cutting side decreasing to 16,053 με, while the peak strain on the working face side remained at approximately 14,643 με. As the primary roof broke, the DFOS strain peak near the working face advanced towards the direction of progression, though both strain values remained relatively stable.

**Figure 6:**
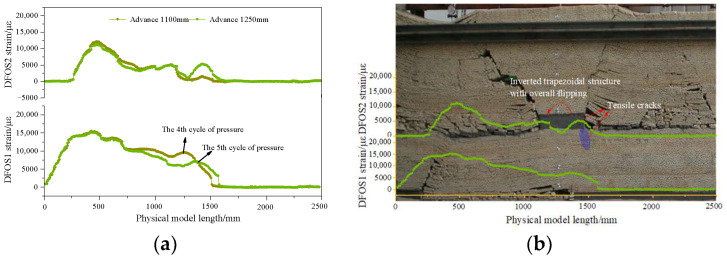
DFOS strain curve before mining through coal pillar. (**a**) Advance from 110 to 1250 mm; (**b**) Overlying rock collapse characteristics when advancing 1250 mm.

The DFOS strain curve and collapse characteristics of the overlying rock after passing through the coal pillar are illustrated in Figure 6. As the working face advances, the inverted trapezoidal structure of the overlying rock above the remaining coal pillar deflects toward the goaf, inducing advanced tension cracks in the floor of the coal pillar ahead of the working face. DFOS2 registers a peak tensile stress at the floor tension crack site with a maximum tensile strain of 1543 με. As the working face progresses, the peak tensile strain of DFOS2 gradually increases to 5319 με, indicating further development of tension cracks at the advanced working face position. Hence, the peak tensile stress in horizontal optical fibers can be employed to characterize the propagation of rock fractures.

**Figure 7:**
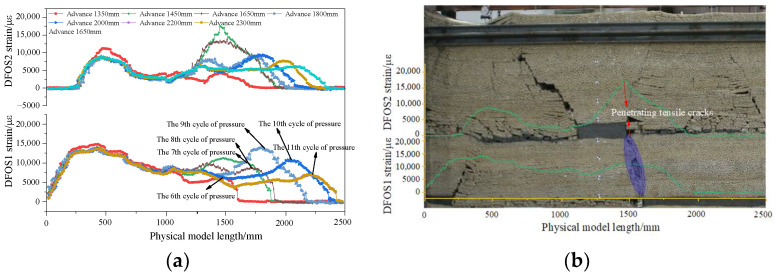
DFOS strain curve after mining through coal pillar. (**a**) Advance from 1350 to 2300 mm; (**b**) Overlying rock collapse characteristics when advancing 1450 mm.

The DFOS strain curve and collapse characteristics of the overlying rock after traversing the coal pillar are depicted in Figure 7. As the working face progresses, the tensile cracks in the floor of the remaining coal pillar continue to expand downward toward the top plate of the coal seam working face. When the working face advances to 1450 mm, the cracks below the remaining coal pillar reach the goaf of the lower coal seam, and the peak tensile strain of DFOS2 attains its maximum value of 17,543 με. At this time, the working face experiences the most severe pressure. The overall subsidence of the inverted trapezoidal overlying rock structure results in the gradual closure of the remaining coal pillar bottom tension cracks, and the peak tensile strain of DFOS2 gradually decreases. As the goaf is compacted, the peak tensile stress of DFOS2 tends to stabilize.

## 5. Characterization of Stress Evolution Law of Overlying Rock Using FBG

### 5.1. FBG Monitoring Analysis

**Figure 8:**
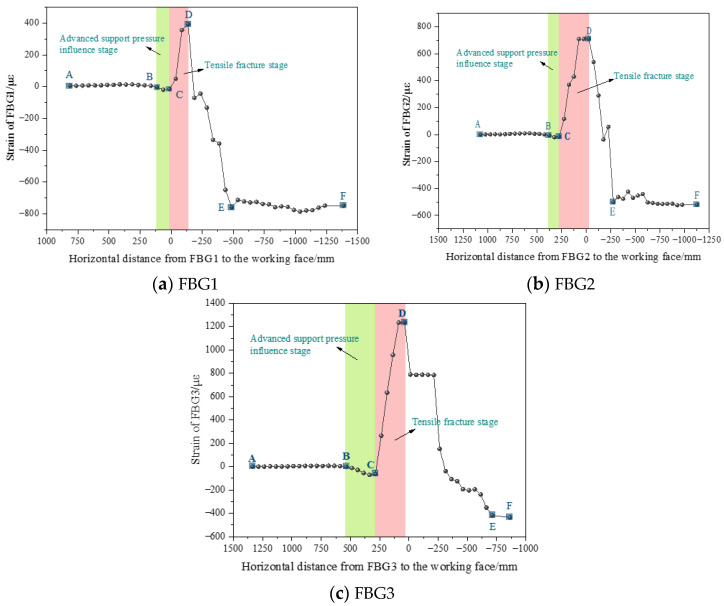
FBG strain changes during the process of advancing the working face.

During the experiment, fiber Bragg grating sensors were employed to monitor the internal strain of the rock layer, and the strain varied with the advancement of the working face, as depicted in Figure 8. The corresponding relationship between the position of the fiber optic grating and the remaining coal pillar indicates that FBG1, FBG2, and FBG3 correspond to the roof strain of coal seams 1^−2^, respectively. The strain curve of the optical fiber grating on the working face passing through the remaining coal pillar displays a generally consistent pattern divided into five stages. As the working face progresses, the roof away from the working face is in the original rock stress stage (AB), and the strain remains unchanged. The BC curve represents the stage of advanced support pressure influence. As the working face approaches the fiber Bragg grating, the top plate in front of the working face is affected by advanced support pressure, and the fiber Bragg grating is compressed negatively, exhibiting an unimodal shape. The rock layer where the fiber optic grating is located collapses toward the goaf under its weight, and the asynchronous collapse of different rock layers from bottom to top causes the fiber optic grating to be subjected to positive tension, entering the tension fracture stage (CD). As the tension cracks gradually expand upward, the tensile strain of the fiber Bragg grating steadily increases until the upper rock layer fractures and collapses. Then, the collapsed rock layer descends toward the goaf under its weight, and the fiber optic grating shifts from tension to sustained compression, entering the sustained compression stage (DE). As the load on the collapsed overlying rock increases, the compressive strain value of the fiber optic grating gradually increases. With the collapse and compaction of the overlying rock, the EF stage signifies the compression stabilization stage (EF) of the goaf rock mass, and the strain of the fiber optic grating gradually tends to stabilize.

**Figure 9:**
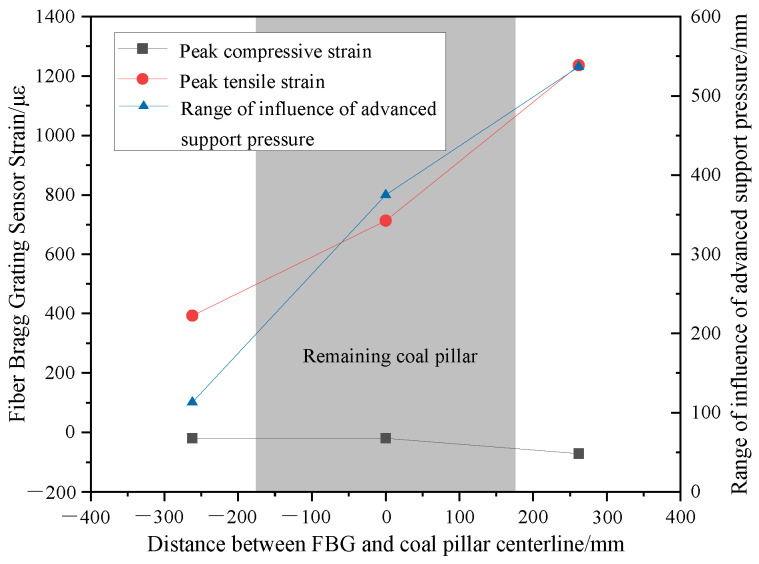
FBG test results and their corresponding relationship with the position of the remaining coal pillar.

The change in strain values of fiber Bragg grating sensors can reflect the stress state of the rock mass at a given location. The distance from the working face, corresponding to the occurrence of FBG compressive strain in front of the working face, indicates the range of influence of advanced support pressure. The peak tensile stress of FBGs can reflect the severity of overlying rock collapse at this location. Figure 8 shows the peak values of compressive strain, the range of influence of advanced support pressure, and the peak values of tensile strain for different FBGs, whereas Figure 9 illustrates their corresponding relationships with the spatial position of the remaining coal pillar. The influence range of the advanced support pressure at the 2^−2^ coal seam working face is 113 mm in front of the remaining coal pillar, and the peak compressive strain is −20 με. The range of pressure influence and peak compressive strain of the advanced support on the working face continue to increase despite passing through the remaining coal pillar. This increase occurs as the overlying rock left of the remaining coal pillar sinks toward the goaf, reducing its support force for the inverted trapezoidal overlying rock structure above the coal pillar and transferring the load of the inverted trapezoidal overlying rock structure to the rear of the coal pillar. The range of influence of advanced support pressure and peak compressive strain on the working face is 4.8 times and 3.6 times, respectively, before passing through the coal pillar. The peak tensile strain of the top plate gradually increases with the expansion of the remaining coal pillar floor cracks. After the cracks penetrate the top plate of the working face, the tensile strain of the top plate reaches its maximum value.

### 5.2. Comparison of Coal Seam Floor Pressure Sensor Testing

**Figure 10:**
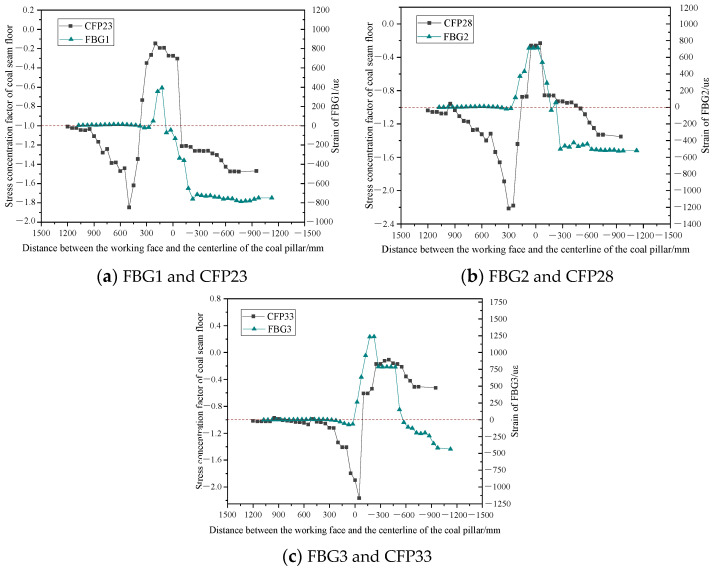
Comparison of monitoring curves between FBG and CFP as the working face advances.

The stress concentration state of the 2^−2^ coal floor during the advancing process of the working face can be monitored in real time through the coal seam floor pressure sensor, which is usually represented by the stress concentration coefficient. When the compressive stress increases, the stress concentration factor increases, a trend that is exactly opposite to that of the FBG strain curve decreasing under compression. The stress concentration factor is set as the ratio of the pressure monitoring value of the coal seam floor pressure sensor at a specific moment to the initial pressure value before mining, multiplied by −1 to maintain the consistency of the force vector. Therefore, when the stress concentration factor equals −1, the floor does not experience stress disturbance. When the stress concentration factor is less than −1, it indicates that the floor pressure increases and vice versa. Figure 10 illustrates the strain curves of FBG1, FBG2, and FBG3 and the stress concentration coefficient curves of coal seam floor pressure sensors CFP23, CFP28, and CFP33 in the process of the working face advancing. It demonstrates that the fiber grating sensor and the coal seam floor pressure sensor perceive changes in overburden stress due to mining at the working face, which are essentially the same. The coal seam floor pressure sensor curve corresponds to the five stages of the original rock stress stage, the advanced support pressure influence stage, the tension fracture stage, the sustained compression stage, and the compression stability stage of the fiber grating strain curve.

The stress disturbance in surrounding rock caused by coal seam mining is transmitted from bottom to top. Hence, the FBG located on the roof of the coal seam experiences a delay in sensing changes in the advanced support pressure of the working face, and energy loss occurs during the upward transmission of stress. However, with the high strain testing accuracy of FBG, a slight change in compressive strain was detected at a position 126 mm above the coal seam when FBG was closer to the working face. Compared to the CFP at the corresponding vertical position, the lag distances of FBG1, FBG2, and FBG3 in sensing the advanced support pressure are 425, 325, and 25 mm, respectively. As the working face mines through the remaining coal pillar, the load of the inverted trapezoidal overburden structure above the remaining coal pillar is transmitted to the 2^−2^ coal seam working face from top to bottom through the remaining coal pillar, reducing the FBGs’ lag in monitoring distance. At this time, the top and floor of the working face are subjected to the overlying rock loads above the 1^−2^ coal pillar and below the 2^−2^ coal pillar, respectively, increasing the stress concentration of the working face during the passage through the remaining coal pillar. After the subsidence and stabilization of the overlying rock, both FBG and CFP monitoring curves tend to stabilize under compression, with compressive stress values higher than before mining.

## 6. Conclusions

This study applies optical fiber monitoring technologies, FBG and BOTDA, to a physical model experiment. The characteristics of overburden movement, working face pressure, and optical fiber strain monitoring curves are analyzed, and the relationship between different characteristics is clarified. The main conclusions are as follows:

(1) The patterns of overlying rock fracture and working face pressure when the lower coal seam working face passes through the remaining coal pillar are obtained, and the patterns are divided into three stages. The fracture angle of the overlying rock near the working face decreases gradually as the mining passes through the coal pillar and the post-passing stage. The mining leads to the formation of a penetrating fracture between the overlying rock of the lower coal seam and the goaf of the upper coal seam, with the caving angle of the penetrating fracture of the overlying rock reaching 75°. The cycle pressure step distance of the working face during mining through the coal pillar is 33.3% lower than that without the influence of the coal pillar.
(2) The horizontally distributed optical fiber characterization of working face pressure is proposed. The peak of fiber optic tensile strain is a manifestation of crack development after horizontal rock fracture, and the peak value of tensile strain increases with the increase in mining-induced crack opening. The strain pattern of the horizontal optical fiber is unimodal when initially placed on the working face and transitions from unimodal to bimodal during periodic pressure. Simultaneously, the peak point of the fiber on one side of the working face advances in the direction of the old top breaking.
(3) The strain curve of fiber Bragg grating can characterize the stress state of overlying rock. During the mining process of the working face passing through the remaining coal pillar, the variation pattern of the fiber optic grating strain curve remains consistent, undergoing stages of original rock stress, advanced support pressure influence, tensile fracture, continuous compression, and stable compression. The accuracy of the fiber optic grating characterization method is verified by comparing it to the monitoring curve of the coal seam floor pressure sensor.
(4) The influence of the remaining coal pillar on the advanced support pressure of the working face is examined. The advanced support pressure value and its influence range continue to increase when the mining face passes through the remaining coal pillar. The advanced support pressure value and its influence range increase, registering 4.8 times and 3.6 times higher than before passing through the remaining coal pillar. The stress concentration rises after passing through the remaining coal pillar on the working face. Further verification of the conclusions of the physical model experiment is necessary combined with engineering experiments.

## Figures and Tables

**Table 1:** Mass ratios of similar materials corresponding to different lithology.

Lithology of Simulated Stratum	Accumulated Thickness (mm)	Mass Ratio (River Sand: Binding Material)	Mass Ratio (Gypsum: Mica Powder)	Sand Mass (kg)	Binding Material Mass	
					Gypsum Mass (kg)	Mica Powder Mass (kg)
Loess	58	9:1	2:8	13.00	0.29	1.16
Medium-grained Sandstone	246	8:1	2:8	69.00	1.73	6.90
Siltstone	418	7:1	2:8	123.60	3.53	14.13
Fine-grained Sandstone	203	8:1	3:7	70.80	2.66	6.20
Coarse-grained Sandstone	19	7:1	2:8	16.00	0.46	1.83
Mudstone	44	9:1	2:8	18.00	0.40	1.60

**Table 2:** The pressure step distance and overburden collapse angle in the process of working face advancement.

Working Face Pressure	Advancing Distance (mm)	Pressure Step Distance (mm)	Collapse Height (mm)	Collapse Angle (°)
Initial pressure	500	500	157	49
The 1st cycle to pressure	650	150	168	61
The 2nd cycle to pressure	850	200	296	56
The 3rd cycle to pressure	1000	150	296	54
The 4th cycle to pressure	1100	100	296	69
The 5th cycle to pressure	1250	150	296	52
The 6th cycle to pressure	1350	150	296	51
The 7th cycle to pressure	1450	150	296	75
The 8th cycle to pressure	1650	200	296	51
The 9th cycle to pressure	1800	150	296	58
The 10th cycle to pressure	2000	200	296	60
The 11th cycle to pressure	2200	200	296	68
The 12th cycle to pressure	2300	100	296	63

## Data Availability

All the data, models, or codes that support the findings of this study are available from the corresponding author upon reasonable request.
